# Effect of Ionic and Non-Ionic Surfactants on the Pasting Characteristics and Digestive Properties of Regular and Frozen Starch for Oral Delivery

**DOI:** 10.3390/foods11213395

**Published:** 2022-10-27

**Authors:** Yan-Bin Tan, Jie-Ying Wei, Yi-Fan Tang, Yu-Tong Ye, Lei Wang, Li-Jun Yang, Zhong-Xiu Chen

**Affiliations:** Molecular Food Science Laboratory, College of Food and Biology Engineering, Zhejiang Gongshang University, Hangzhou 310018, China

**Keywords:** starch, emulsifiers, gelatinization, digestion

## Abstract

Starch is an ideal wall material for controlled release in oral delivery systems due to its non-allergic properties, availability, and cheap price. However, because of its poor mechanical behavior and high water permeability, it is necessary to modify the amphiphilic nature of starch. Surfactants are essential components to emulsify the lyophobic food ingredients. However, the interaction of starch with emulsifiers and how they affect the pasting behavior and digestion of starch are not well understood. In this paper, surfactants, such as non-ionic Tween (TW) and ionic sodium fatty acid (NaFA), with varying hydrophobic carbon chain lengths, were selected as model amphiphiles to investigate the structural, pasting, rheological properties and in vitro digestibility of regular and frozen starch samples. The results showed that, in most cases, the addition of TW reduced the viscosity of starch. However, saturated medium-chain NaFA increased the starch viscosity and rheological modulus greatly. Both surfactants inhibited starch digestion. This paper presents a comparative investigation on the effect of ionic and non-ionic surfactant on the structure and properties of corn starch, and therefore the information is useful for structural-based formulation with starch for developing colloidal delivery systems. It is also helpful for developing functional food with controllable digestion properties.

## 1. Introduction

Starch is one of the most abundant natural carbohydrate polymers. It is an ideal wall material for the controlled release in oral delivery systems due to its non-allergic properties, availability and low-cost [1]. The starch-based materials are hydrophilic, which increases the degradation rate. However, their poor mechanical behavior and the high water vapor permeability limit their applications for controllable release or digestion. Modifying the amphiphility of starch is an effective strategy to solve these drawbacks [2]. Surfactants are essential components to emulsify the lyophobic food ingredients such as fat-soluble vitamins, flavors, polyphenols, carotenoids, and probiotics. In a controlled release delivery system, the stability of the embedded substances and their digestion and absorption process are important characteristics. However, the modification of starch with emulsifiers and how they affect the starch pasting and digestion should also not to be ignored. The properties and digestion of starch depend on its molecular structure and self-assembled semicrystalline nature. Methods of changing the starch structure include chemical derivation and physical modification. Chemical derivation focuses on the synthesis of various starch esters [3]. Physical modification which involves non-covalent interactions between starch and fatty acids [4], flavors [5] or phenolic compounds [6] could alter the starch properties and digestibility. Food surfactants have both hydrophilic groups available for hydration and hydrocarbon chains for hydrophobic interactions, which are often used in the emulsification of lipids due to their ability to reduce the surface tension. From a structural point of view, the hydrophobic chain of a surfactant is able to intervene in the assembled structure of the starch through hydrophobic interactions. Additionally, if the surfactant is charged, the hydration of the ions cannot be ignored. Emulsifiers that are widely used in oral delivery systems are nonionic surfactants. For example, gelatinization and rheological properties of lotus seed starch could be modified by complexation with glycerine monostearate [7]. Polysorbates could form complexes with starch, which caused the reduced digestibility of starch [8]. In addition to research on surfactant-dependent interactions in starch nanoparticles preparation [9], there have been few systematic studies investigating the influence of the charged group of ionic surfactants on the gelatinization process and the digestion properties of starch. Therefore, this paper will focus on the comparative study of the influence of ionic and nonionic amphiphiles on the structure and properties of starch. It is known that temperature could affect the reassociation or the recrystallization of the amylase and amylopectin in gelatinized starch, and thus freezing might affect the retrogradation of starch [10]. Moreover, freezing is one of the most effective preservation technologies for food and is sometimes involved in the development of oral drug delivery systems. The effects of starch sources [11], freezing temperatures [12], and freezing methods [13] on the molecular, crystal, physicochemical properties and digestibility of starch have been reported. Additives, such as sugars [14] and salt [15], have been found to change the structure of frozen starch. However, how food emulsifiers affect the structure and property of frozen starch remains unclear. Theoretically, the hydrophobic interaction is entropy-driven and thus intrinsically temperature-sensitive [16], while the freezing process may affect the assembly of starch, thus affecting its interaction with the surfactant, its rheological properties, and the efficiency of enzymatic hydrolysis on starch pastes. In this study, both regular and frozen starch were used as the starting materials and non-ionic Tween (TW) and ionic surfactant, sodium fatty acid (NaFA) with varying hydrophobic carbon chain lengths, were selected as model surfactants to treat starch. The effects of ionic and nonionic emulsifiers on starch were investigated, while the structure-dependence on the pasting and digestion of starch were summarized. Because there have been few systematic studies on the influence of ionic surfactants on starch, this paper presents a comparative investigation on the effect of ionic and non-ionic surfactants on the structure and properties of corn starch. The results are useful for understanding the interaction between amphiphilic additives and starch in developing stable oral delivery systems, and are helpful for functional food formulation for controllable digestion.

## 2. Experimental Work

### 2.1. Materials

Corn starch was purchased from Shanghai Aladdin Biochemical Technology Co., Ltd. (Shanghai, China). Tween 20 (polyethylene glycol sorbitan monolaurate), Tween 40 (polyoxyethylene sorbitan monopalmitate), Tween 60 (polyethylene glycol sorbitan monostearate) and Tween 80 (polyethylene glycol sorbitan monooleate) (Abbreviated as TW20, TW40, TW60 and TW80 respectively), sodium n-caprylate (NaFA (C-8)), sodium capricate (NaFA (C-10)), sodium laurate (NaFA (C-12)) and sodium oleate (NaFA (C-18)) were purchased from TCI Corporation. α-Amylase from porcine pancreas was purchased from Sigma-Aldrich (Shanghai, China).

### 2.2. Preparation of Frozen Starch

Corn starch suspension (6 *w*/*v*%) was obtained by adding corn starch into ultrapure water. The starch suspension was gelatinized in a boiling water bath for 20 min and cooled to room temperature. After it had been frozen in a refrigerator at −18 °C for 24 h, the starch was taken out and melted in a water bath at 25 °C for 3 h. The obtained starch gel was then freeze-dried before subsequent measurement.

### 2.3. Pasting Properties

The pasting properties of starch were measured by using a Rapid Visco Analyzer with paddle agitator (RVA, Tech Master, Perten Instruments, Hägersten, Sweden). The method was referred to by Zheng et al. [17], with slight modifications. Corn starch was mixed with ultrapure water in aluminum RVA canisters to make a 6% (*w*/*w*) starch mixture, and then surfactants were added to it. Surfactant-free corn starch was used as a control. The starch suspension was maintained at 50 °C for 1 min and heated to 95 °C (12 °C/min) for 2.5 min. The mixture was then cooled to 50 °C at 12 °C/min and held for 2 min. The pasting temperature, peak viscosity, breakdown, setback, and final viscosity was recorded throughout the process. After gelatinization, the starch paste was freeze-dried, ground into a powder with a mortar, and stored at 4 °C for structural characterization.

### 2.4. Dynamic Rheological Measurements

Dynamic rheological measurement was carried out by a rheometer (AR-G2, TA Instruments, New Castle, DE, USA). The gelatinized starch gel obtained in the RVA test was transferred to the test platform at 25 °C. The frequency sweep was set to a range of 0.1–10 Hz at 1% strain (within the linear viscoelastic region). The gap was set at 1 mm. The elastic modulus (G′) and viscous modulus (G″) were obtained by TA rheometer data analysis software (TA Instruments, New Castle, DE, USA).

### 2.5. Fourier Transform Infrared (FTIR) Spectroscopy

The starch samples used for FTIR spectroscopy were obtained by freeze-dried products from RVA. The sample (10 mg) and KBr (200 mg) were ground and pressed into sheets for the FTIR measurement (iS50 FT-IR, Thermo Fisher Scientific Inc., Waltham, MA, USA). Spectra were recorded from 4000 to 400 cm^−1^. The number of scans was 48, and the resolution was 4 cm^−1^. FTIR data were analyzed with OMNIC software (Thermo Fisher Scientific Inc., Waltham, MA, USA). 

### 2.6. In Vitro Digestibility

The in vitro enzyme digestibility of starch was determined by 3, 5-dinitrosalicylic acid (DNS) colorimetry [18]. Five hundred mg of corn starch or frozen starch was added with 50 mL of phosphoric acid buffer (10 mmol·L^−1^, pH = 7.0), and the mixture was gelatinized in a boiling water bath for 20 min. The obtained starch solution (10 mg/mL) was cooled to 37 °C and used as the stock solution.

The surfactant (40 mg) was mixed with starch solution (8 mL) and 12 mL phosphoric acid buffer. The obtained solution was placed in a water bath pot at 37 °C and stirred magnetically for 30 min at 300 rpm. α-Amylase was treated by centrifugation for 20 min (4 °C, 8000 rpm). Next, 1 mL supernatant of α -amylase (0.19 mg/mL) was added to the above starch-surfactant mixture to start the enzymatic reaction.

At a specific interval (0, 1, 5, 10, 30, 60, 120 and 180 min), the product was taken out and added with DNS, and then reacted in a boiling water bath for 3 min. After being rapidly cooled by ice water, the reaction mixture was diluted with ultrapure water. The content of reducing sugar content was calculated according to the absorbance at 540 nm, which was determined with a microplate reader.

### 2.7. Statistical Analysis

Each experiment was repeated three times. A statistical analysis was performed using Origin 2022b. One way Duncan analysis (SPSS-21.0) was used for the significant difference test, and the results were reported as means and standard deviations.

## 3. Results and Discussion

### 3.1. The Pasting Properties of Corn Starch in the Presence of TW and NaFA

**Figure 1:**
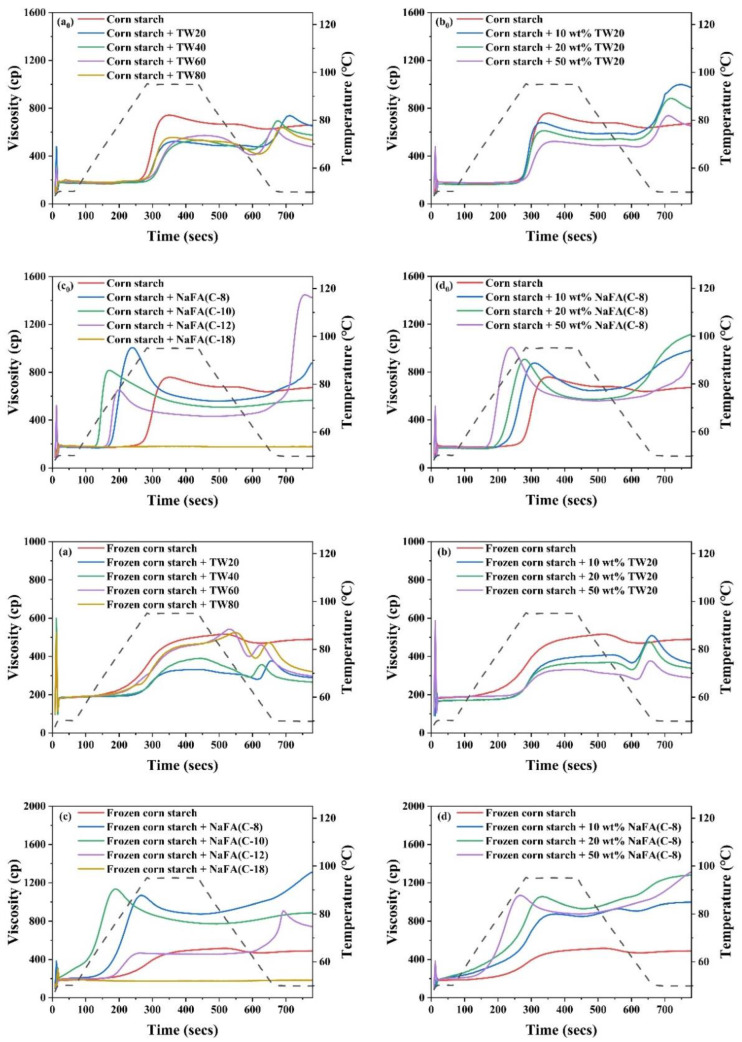
RVA pasting curves of regular (**a_0_**–**d_0_**) or frozen (**a**–**d**) corn starch in the presence of Tween (TW) and sodium fatty acid (NaFA). (**a_0_**,**a**) RVA curves in the presence of different types of TW (50 wt%). (**b_0_**,**b**) RVA curves in the presence of different concentrations of TW20. (**c_0_**,**c**) RVA curves in the presence of different types of NaFA (50 wt%) (**d_0_**,**d**) RVA curves in the presence of different concentrations of NaFA (C-8).

**Figure 2:**
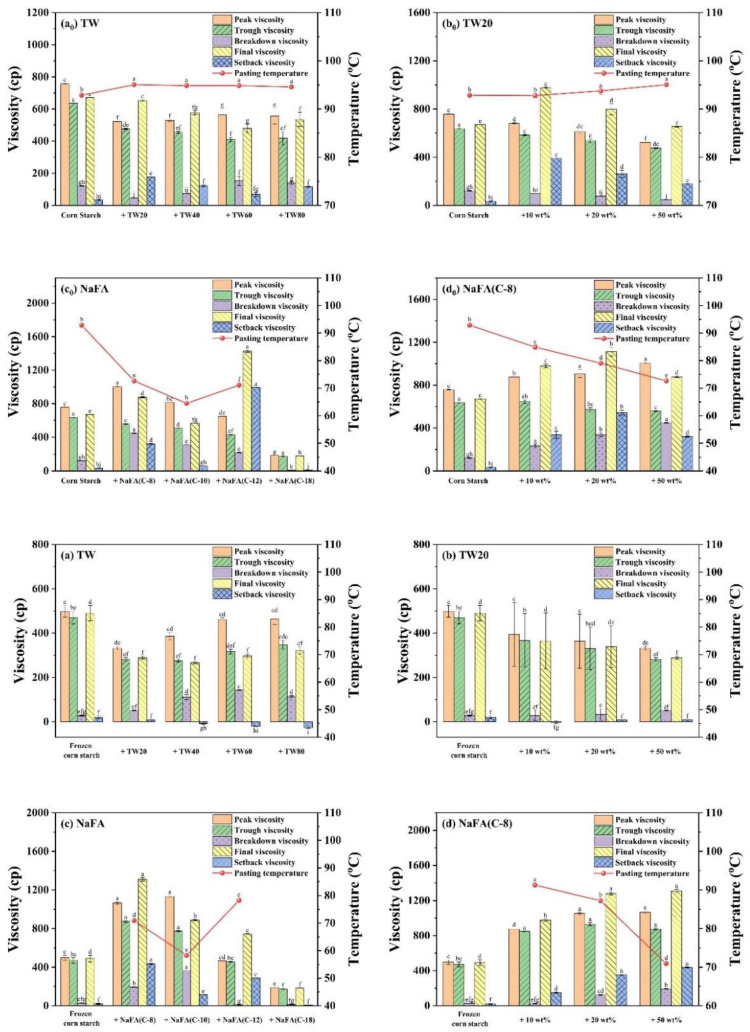
Pasting parameters (viscosity and pasting temperature derived from RVA curves) of regular or frozen corn starch in the presence of Tween (TW) and sodium fatty acid (NaFA). (**a_0_**,**a**) Pasting parameters in the presence of different types of TW (50 wt%). (**b_0_**,**b**) Pasting parameters in the presence of different concentrations of TW20. (**c_0_**,**c**) Pasting parameters in the presence of different types of NaFA (50 wt%). (**d_0_**,**d**) Pasting parameters in the presence of different concentrations of NaFA (C-8). Means with different letters differed significantly at *p* < 0.05.

Starch pasting occurs after heating starch above the pasting temperature. Consequently, the starch granules lose their crystalline structure, absorb water, swell, and become viscous. Figure 1a_0_–d_0_ shows the RVA pasting curves of corn starch in the presence of TW and NaFA surfactants. The pasting parameters obtained from the RVA curves are shown in Figure 2. The peak viscosity of the RVA curve during starch pasting is related to the solvation process and swelling capacity of starch granules [19]. As shown in Figure 1a_0_, after the addition of TW, the peak viscosity of corn starch decreased. As the length of the hydrophobic chain of TW20, TW40, and TW60 increased, the peak viscosity showed a slight increase. Figure 1b_0_ shows that as the concentration of TW20 increased, the peak viscosity of starch decreased. NaFA is an ionic surfactant in which the head group undergoes strong hydration. Therefore, the interaction of its hydrophobic groups with the starch hydrophobic domain, as well as the competitive hydration of its polar group, jointly affected the gelatinization of starch. Medium-chain fatty acids, such as NaFA (C-8) and NaFA (C-10), promoted the hydration of the starch chain, which was manifested by an increase in peak viscosity (Figure 1c_0_). In Figure 1d_0_, as the concentration of NaFA (C-8) increased, the peak viscosity of starch increased. However, within the range of several fatty acids examined, the viscosity decreased with an increase in chain length. This is different from the case of starch with TW, suggesting different hydrophobic chain-length dependence effects of ionic and nonionic surfactants. It is noteworthy that no viscosity peak was observed in starch with sodium oleate (C18). Wang [20] et al. found that the fatty acid with 14 or more carbon units did not show a viscosity peak during the RVA cooling stage. They proposed that the longer fatty acids chain is less liable to form complexes with starch because long-chain fatty acids tend to self-aggregate in aqueous systems when the concentration exceeds the critical micellar concentration (C.M.C.). In this work, the concentration of surfactants used exceeded the C.M.C. The results of a comparative study of TW and NaFA suggest that NaFA’s hydrophobic interaction with starch, as well as the competitive hydration of its polar head group, affected the hydration of starch, thereby changing the pasting viscosity of starch.

As shown in Figure 2, the addition of TW slightly increased the pasting temperature of corn starch, indicating that the swelling of starch granules was delayed. The possible reason is that an insoluble layer formed between partially leached amylose and surfactants on the surface of the starch granules, thereby hindering the transport of water to the particles and delaying the expansion of starch particles [21]. The addition of NaFA significantly reduced the pasting temperature of corn starch, possibly due to the presence of salt ions in the system.

During the cooling stage (95 °C to 50 °C), a viscosity peak was observed in the TW system. It was suggested that the viscosity peak at cooling is associated with the formation of starch-surfactant complexes [20,22]. The viscosity peak decreased with the increase of the carbon chain length, indicating that TW with medium carbon chains was more likely to form a complex with corn starch, which is consistent with the literature [23]. The final viscosity of the corn starch-TW paste was lower than that of the pure starch, and increased as the length of the TW carbon chain decreased. For starch with sodium laurate (NaFA-C12), a high viscosity peak was observed during the cooling stage, which is consistent with the effect of fatty acids on wheat starch [24]. In general, starch paste mixed with medium-chain NaFA exhibits an increase in the final viscosity. TW and NaFA behaved differently on regulating the property of the peak viscosity, pasting temperature and the final viscosity of starch.

Compared with regular corn starch, the frozen corn starch shows an irregular RVA curve and lower peak viscosity, trough viscosity and final viscosity (Figure 1a–d). After the addition of TW, the increased peak viscosity was hydrophobic-chain-length dependent. TW reduced the pasting viscosity of frozen starch. The reason may be that TW could form a complex with the dispersed amylose and adhere to the surface of the starch particles, so it might inhibit the hydration of the starch, which generally manifests as a decrease in viscosity [8,25]. The addition of medium-chain fatty acids, such as NaFA (C-8) and NaFA (C-10), greatly increased the peak viscosity of frozen starch, and as the concentration of NaFA (C-8) increased, the peak viscosity increased. The final viscosity indicates the ability of starch to form a viscous paste or gel after heating and cooling [26]. Starch pastes with the NaFA (except NaFA (C-18)) have a higher final viscosity than frozen starch, and shows a chain length and concentration dependence. Compared to unfrozen starch, the pasting temperature of frozen starch was not observed, even in the presence of TW. The reason is not very clear. However, in the presence of NaFA, it shows a lower pasting temperature than unfrozen corn starch, which indicates that lower energy was needed for surfactant-frozen starch mixtures. During the cooling stage (95 °C–50 °C), a viscosity peak of the frozen starch paste was observed in the presence of TW. For NaFA (C-18), no viscosity peak was observed, suggesting that surfactants with longer chains were less likely to form complexes with starch. Taken together, the peak viscosity of starch or frozen starch increased with the increase of TW carbon chain length and decreased with the NaFA carbon chain length. Furthermore, the peak viscosity decreased with the increase of TW20 concentration but showed the opposite concentration dependence in the case of NaFA (C-8). Whether the starch was frozen or not, TW reduced the peak viscosity of the starch, while medium-chain NaFA increased the peak viscosity of the starch.

### 3.2. Effects of TW and NaFA on Rheological Properties of Corn Starch

**Figure 3:**
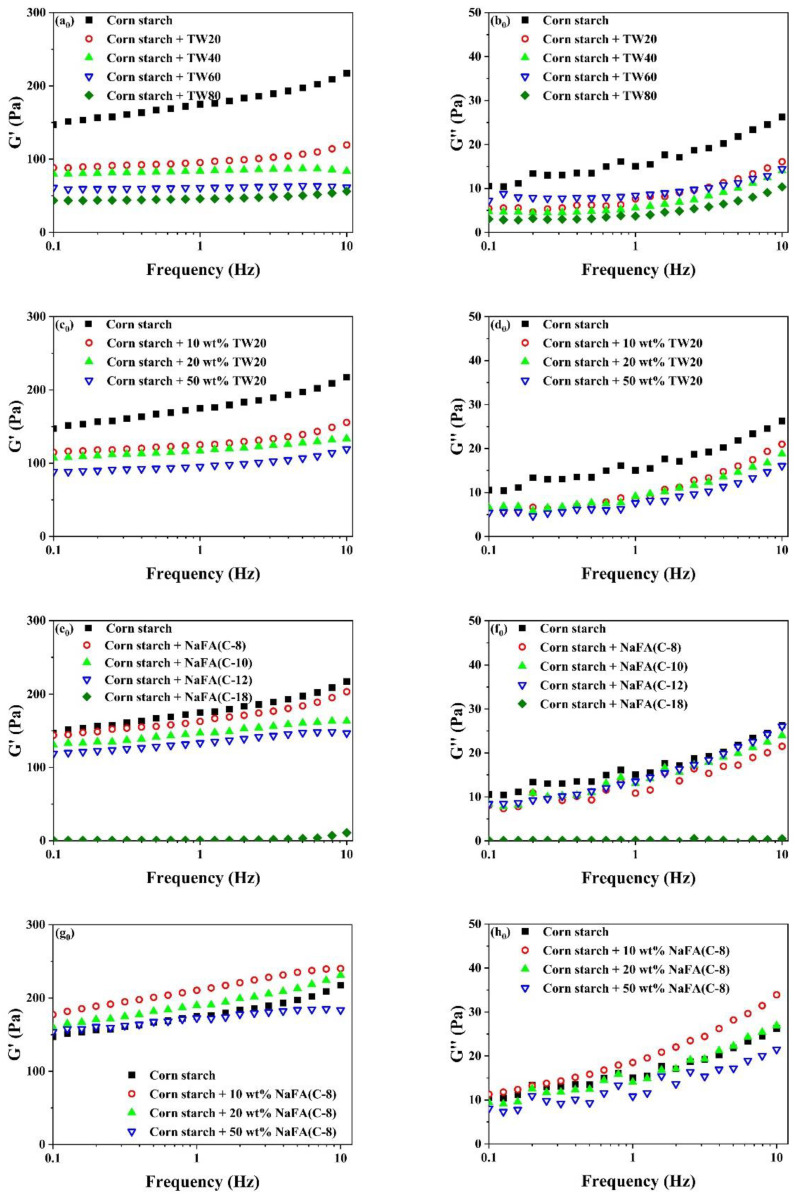

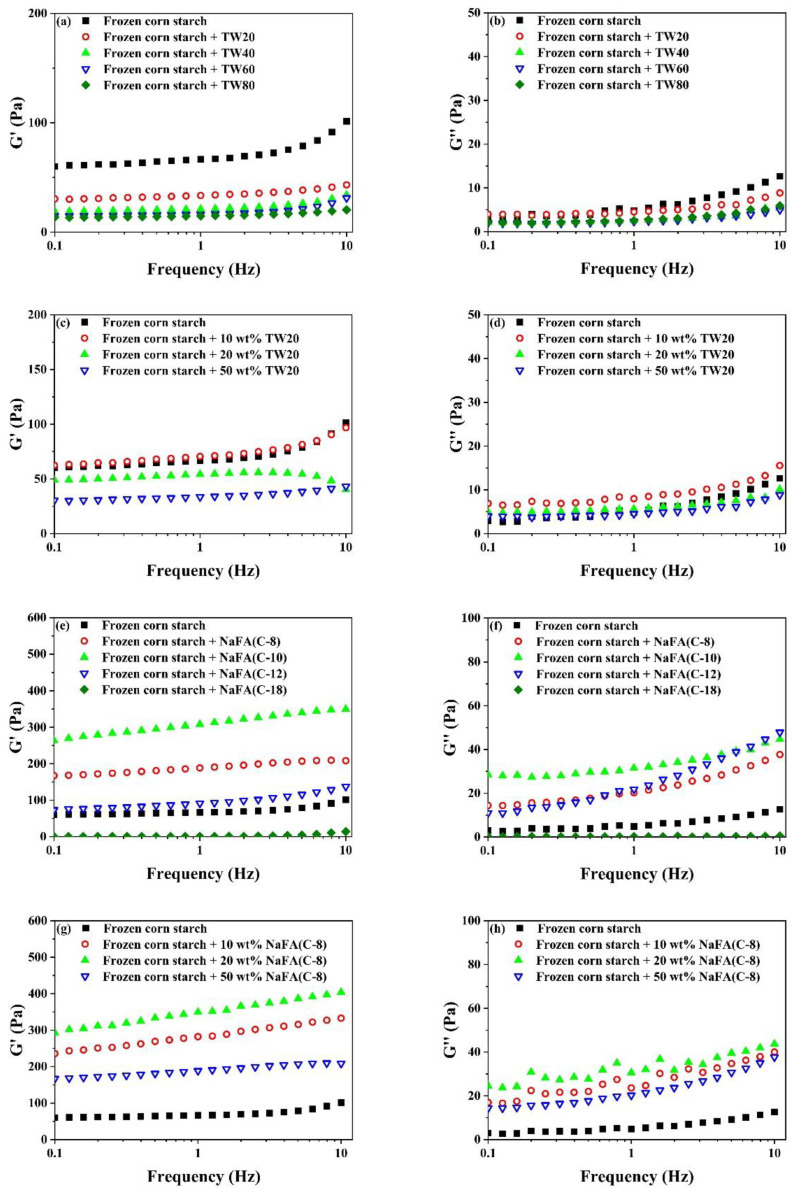
Rheological properties of regular (**a_0_**–**h_0_**) or frozen (**a**–**h**) corn starch in the presence of Tween (TW) and sodium fatty acid (NaFA). (**a_0_**,**a**) Elastic modulus (G′) of corn starch in the presence of different types of TW (50 wt%). (**b_0_**,**b**) Viscous modulus (G″) of corn starch in the presence of different types of TW(50 wt%). (**c_0_**,**c**) G′ of corn starch in the presence of different concentrations of TW20. (**d_0_**,**d**) G″ of corn starch in the presence of different concentrations of TW20. (**e_0_**,**e**) G′ of corn starch in the presence of different types of NaFA (50 wt%). (**f_0_**,**f**) G″ of corn starch in the presence of different types of NaFA (50 wt%). (**g_0_**,**g**) G′ of corn starch in the presence of different concentrations of NaFA (C-8). (**h_0_**,**h**) G″ of corn starch in the presence of different concentrations of NaFA (C-8).

Rheological properties of unfrozen or frozen corn starch in the presence of TW and NaFA are shown in Figure 3. It can be seen that the elastic modulus (G′) was greater than the viscous modulus (G″) over the entire scanning range, suggesting that the corn starch-surfactant mixture showed a typical weak gel. Figure 3a_0_–h_0_) shows that the addition of TW or NaFA lowered the G′ of the starch paste. As the length of the TW carbon chain or the concentration of TW20 increased, both G′ and G″ decreased gradually. This may be due to the fact that the surfactant prevented the amylose molecules in the starch particles from precipitating into the starch paste and inhibited the expansion of the starch granules [27].

Figure 3a–h shows that both G′ and G″ decreased after starch was frozen. The addition of TW led to a further reduction in G′ and G″. Both G′ and G″ decreased as the carbon chain length or concentration of TW increased. The reduction of G′ and G″ of starch gels confirms that surfactants in the starch network prevented or slowed down the recombination of amylose molecules during cooling. This is consistent with the rheological properties of unfrozen starch. It is worth mentioning that the effect of NaFA on the rheological properties of frozen starch is different from that of TW. NaFA (C-8) and NaFA (C-10) resulted in a significant increase in G′ and G″ of frozen starch, and no chain length and concentration dependence effects of G′ was observed. This result shows that the saturated NaFA with a medium-chain could promote the formation of the network of frozen starch and increase G′ greatly. This is consistent with the results obtained by the RVA. However, unsaturated NaFA (C-18) caused the corn starch paste to form an unstable colloidal system in which G′ and G″ are close to zero. Previous studies also have found that potato starch pastes with high concentration surfactants have similar non-gelling behavior [28].

In summary, in terms of rheological properties, the G′ of unfrozen starch decreased with the increase of the carbon chain length of TW and NaFA, while the G″ of starch remains unchanged in the presence of NaFA. As the length of the hydrophobic carbon chain of TW increases, G″ decreases regularly. For frozen starch, medium-chain NaFA (C-8) at lower concentrations and NaFA (C-10) increased the G′, while TW and long-chain NaFA reduced G′.

### 3.3. Effects of TW and NaFA on the Medium-Range Ordered Structure of Corn Starch

**Figure 4:**
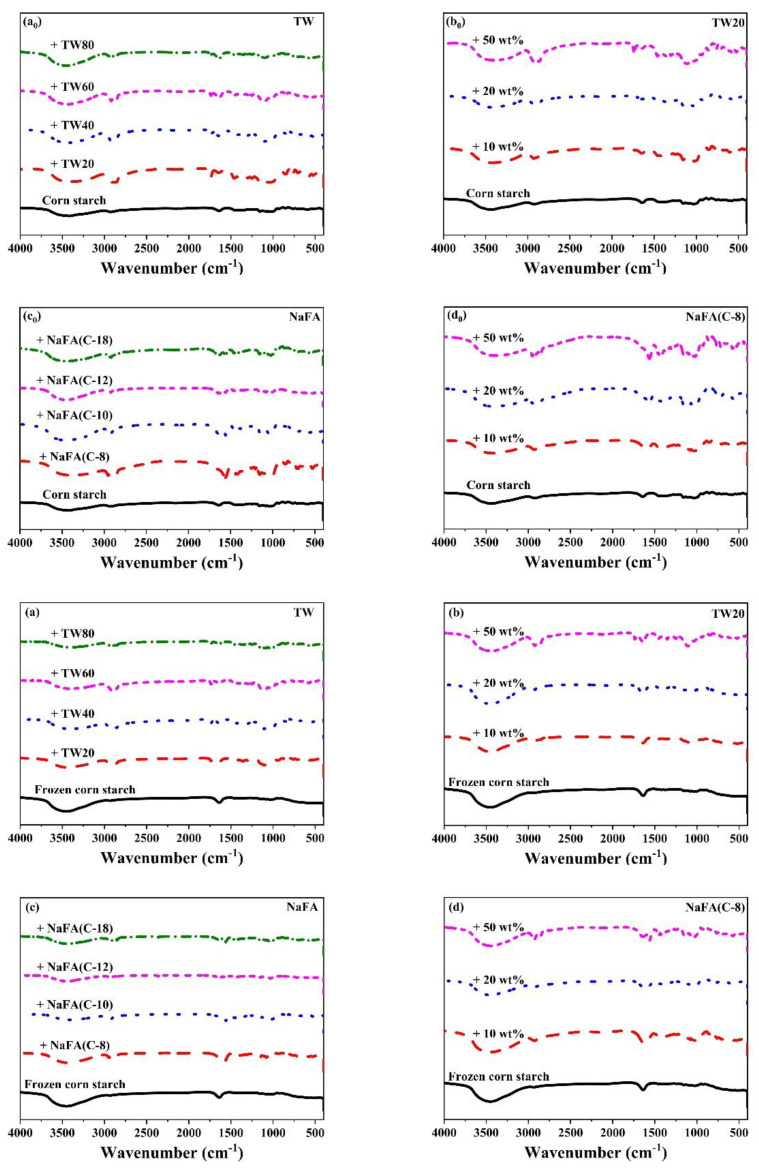
FT-IR spectra of regular (**a_0_**–**d_0_**) or frozen (**a**–**d**) corn starch in the presence of Tween (TW) and sodium fatty acid (NaFA). (**a_0_**,**a**) FT-IR spectra of corn starch in the presence of different types of TW (50 wt%). (**b_0_**,**b**) FT-IRspectra of corn starch in the presence of different concentrations of TW20. (**c_0_**,**c**) FT-IR spectra of corn starch in the presence of different types of NaFA (50 wt%). (**d_0_**,**d**) FT-IR spectra of corn starch in the presence of different concentrations of NaFA (C-8).

Figure 4 shows the FT-IR spectra of corn starch in the presence of TW and NaFA. The band between 3500–3100 cm^−1^ is ascribed to the stretch vibration of the hydroxyl group [29]. For frozen or unfrozen starch, after the addition of TW or NaFA, the peak ascribed to the hydroxyl group was narrowed and the strength was enhanced, indicating that the interaction between the starch and emulsifier had occurred. Zhao [30] et al. found that frozen starch appeared to be easier to disperse in aqueous media, resulting in hydrogen bond rearrangement within starch granules [31]. The peak at 2928 cm^−1^ is the stretch vibration of -CH_2_ of the polysaccharide [32] in which the strength is significantly reduced for frozen starch. Peaks around 2900 cm^−1^ are attributed to C-H stretch vibrations. After the addition of surfactants, two new C-H stretching vibrations peaks appeared which comes from the asymmetrical and symmetrical components of CH_2_ and CH_3_ stretching due to the presence of hydrophobic chains of fatty acid. For systems containing TW or NaFA, peak strength at 2900 cm^−1^ shows chain length and concentration dependence. The strong sharp peak at 720 cm^−1^, which originally belonged to NaFA, disappears in the starch-NaFA mixture, possibly because NaFA entered the double helix structure of starch to form a complex.

**Figure 5:**
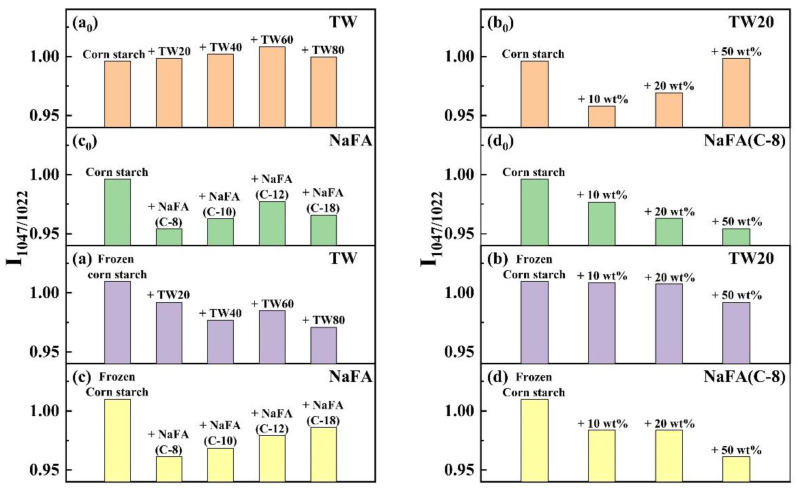
Change of band intensity ratios of regular or frozen corn starch at 1047 cm^−1^ and 1022 cm^−1^ (I_1047_/_1022_) in the presence of Tween (TW) and sodium fatty acid (NaFA). (**a_0_**,**a**) I_1047_/_1022_ of regular or frozen corn starch in the presence of TW (50 wt%). (**b_0_**,**b**) I_1047_/_1022_ of regular or frozen corn starch in the presence of different concentrations of TW20. (**c_0_**,**c**) I_1047_/_1022_ of regular or frozen corn starch in the presence of NaFA (50 wt%). (**d_0_**,**d**) I_1047_/_1022_ of regular or frozen corn starch in the presence of different concentration of NaFA (C-8).

The infrared peak range of 1065–950 cm^−1^ is the fingerprint region that characterizes the conformation changes and ordered double helix structure of the starch [33]. The ratio of peak strength at 1047 to 1022 cm^−1^ (I_1047/1022_) reflects the relative content between the ordered and amorphous structures of starch. Figure 5 shows the I_1047/1022_ of freeze-dried corn starch paste which was mixed with TW or NaFA. For both surfactants, I_1047/1022_ increases as the hydrophobic chain length increases, indicating an increase in the order of medium-range structure. However, for ordinary starch containing TW, the I_1047/1022_ value is higher than pure starch, while the system containing NaFA has a lower I_1047/1022_ value than pure starch. TW with longer chain lengths made the structure of corn starch more orderly, but NaFA reduced the order of structure. TW20 and NaFA exhibited opposite concentration dependence. In the case of frozen starch, I_1047/1022_ is higher than that of unfrozen starch, indicating that the freezing treatment facilitated the orderly arrangement of starch granules and promoted starch retrogradation. Yang [13] et al. also found that freezing may reduce the content of amorphous substances, causing reassembly of starch molecules, which in turn increased the content of medium-range ordered structures. Unlike the ordinary starch, TW with different hydrophobic carbon chains reduced the medium-range orderliness of frozen starch. However, the longer-chain NaFA resulted in a higher content of medium-range ordered structure, which was consistent with the results presented by ordinary starch. These results imply that freezing changes the way TW interacts with starch, while NaFA retains the original mode in the interaction.

### 3.4. Effects of TW or NaFA on the Digestibility of Corn Starch

**Figure 6:**
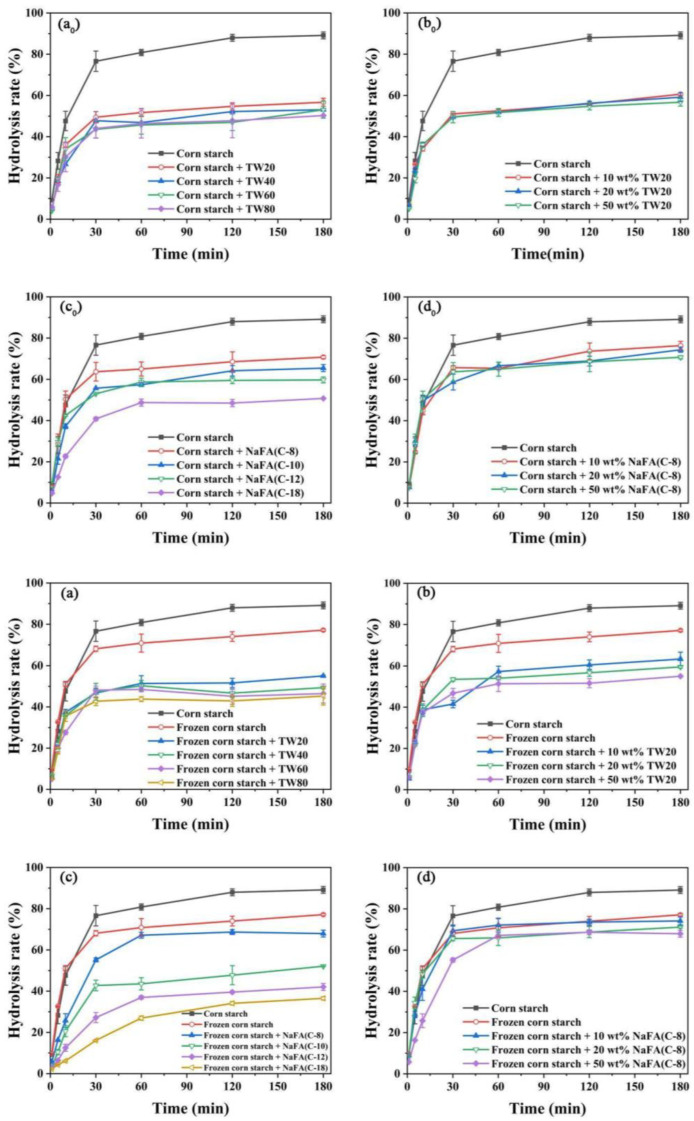
Digestibility of regular (**a_0_**–**d_0_**) or frozen (**a**–**d**) corn starch in the presence of Tween (TW) and sodium fatty acid (NaFA). (**a_0_**,**a**) The effect of carbon chain length of TW on starch digestibility (50 wt%). (**b_0_**,**b**) The effect of concentrations of TW20 on starch digestibility. (**c_0_**,**c**) The effect of carbon chain length of NaFA on starch digestibility (50 wt%). (**d_0_**,**d**) The effect of NaFA (C-8) concentration on starch digestibility.

The above results show that after the addition of TW or NaFA, the pasting properties and microstructure of both ordinary starch and frozen starch were changed. In order to investigate the enzymatic hydrolysis of starch that has been treated with freezing and surfactants, the digestibility of starch was studied. Compared with additive-free corn starch under the same conditions, the pure starch was digested by α-amylase by nearly 90% in 180 min. In the corn starch-NaFA system (Figure 6c_0_), starch digestibility decreased as the length of the hydrophobic chain increased. The final digestibility of starch-NaFA (C-8) was 78.68%, and the final digestibility of the starch-NaFA (C-18) was 50.76%. The effects of both surfactants on starch digestion show chain-length and concentration dependence. Previous studies showed that non-waxy rice starch with long-chain saturated fatty acids had lower in vitro digestibility than those with medium-chain fatty acids added [34]. The decreased in vitro digestibility of corn starch with NaFA may be attributed to the hydrophobic interaction between amylose and fatty acids, which caused the spiral conformation and crystal structure to collapse, thus resisting the attack of enzymes [35]. The formation of amylose-lauric acid complex was a key factor in the reduction of the starch hydrolysis rate [36]. Tween 80 would aggregate on the surface of the starch particles, forming a protective barrier that restricted the entry of enzymes, thereby reducing the digestibility of starch [8].

Figure 6a–d shows the digestibility of the frozen starch after the addition of emulsifiers. Retrogradation of the starch gel occurred during freezing, which increased the content of resistant starch. The complexation of starch with surfactant forms another type of resistant starch [37]. An increase in the content of resistant starch may lead to a further decrease in the digestibility of the frozen starch. Combined with the above, it is concluded that the addition of both TW and NaFA could reduce the digestibility of starch. As the length of the hydrophobic carbon chain of the surfactant increased and the concentration increased, the starch digestibility decreased. Freezing-treating and the addition of TW or NaFA surfactants additively inhibited starch digestion.

## 4. Conclusions

In this study, typical ionic and non-ionic amphiphiles such as Tween (TW) and sodium fatty acid (NaFA) were selected to study their effects on pasting properties, rheological properties, structure and digestibility of regular or frozen corn starch. The results show that NaFA, an ionic surfactant, provided a stronger electrostatic effect, and therefore had a greater effect on starch viscosity than TW. NaFA with a shorter chain may be able to assist in the decrystallization of amylose, resulting in more amylose dissolving and stronger hydration, thus increasing the viscosity of starch in most cases. The effect of NaFA on viscosity was more pronounced than the effect of TW. NaFA and TW have very different effects on the rheological properties of frozen starch. The addition of TW could increase the content of medium-range ordered structure of starch whereas NaFA showed opposite behavior. However, both surfactants could inhibit the digestibility of starch. A certain chain length and concentration dependence effect were observed for both surfactants. This study has significance for understanding the mechanism of using amphiphilic food additives to modify the processing performance and digestibility of starch. Moreover, this paper presents a comparative investigation on the effect of ionic and non-ionic surfactants on the structure and properties of corn starch, and collects useful information for the development of starch-based colloidal delivery systems for control-release purposes.

## Data Availability

The data presented in this study are available on request from the corresponding author.
